# Novel association patterns of cardiac remodeling markers in patients with essential hypertension and atrial fibrillation

**DOI:** 10.1186/1471-2261-11-77

**Published:** 2011-12-28

**Authors:** Andreas S Kalogeropoulos, Sotirios Tsiodras, Angelos G Rigopoulos, Eleftherios A Sakadakis, Andreas Triantafyllis, Dimitrios TH Kremastinos, Ioannis Rizos

**Affiliations:** 1 Department of Cardiology, Hammersmith Hospital, Imperial College Healthcare NHS Trust, London, UK; 2 4th Academic Department of Internal Medicine and Infectious Diseases, University of Athens Medical School, Attikon University Hospital, Athens, Greece; 3 2nd Department of Cardiology, University of Athens Medical School, Attikon University Hospital, Athens, Greece

## Abstract

**Background:**

Matrix metalloproteinases (MMPs) and their tissue inhibitors (TIMPs) are essential for the cardiac extracellular matrix (ECM) remodeling. We investigated differences in serum levels of these markers between patients with atrial fibrillation (AF) and sinus rhythm (SR).

**Methods:**

Serum levels of MMP-2, MMP-3, MMP-9 and TIMP-1 were measured in 86 patients: 27 on SR without any AF history, 33 with paroxysmal and 26 with permanent AF. All subjects had essential hypertension, normal systolic function and no coronary artery disease.

**Results:**

Patients with AF had higher MMP-2, MMP-3 and MMP-9 and lower TIMP-1 compared to SR subjects (all p < 0.001). Paroxysmal AF was associated with higher MMP-2 levels compared to permanent AF (p < 0.001). Matrix metalloproteinase-9 but not MMP-3 was higher in permanent compared to paroxysmal AF group (p < 0.001). Patients with AF had lower levels of TIMP-1 compared to those with SR while permanent AF subjects had lower TIMP-1 levels than those with paroxysmal AF (p < 0.001 for both comparisons). Lower TIMP-1 was the only independent factor associated with AF (OR: 0.259, 95%CI: 0.104-0.645, p = 0.004).

**Conclusions:**

In hypertensives, paroxysmal AF and permanent AF differ with respect to serum MMPs. Increased MMP-2 is associated with paroxysmal, whereas increased MMP-9 with permanent AF. Additionally, lower levels of TIMP-1 had a strong association with AF incidence.

## Background

Atrial fibrillation (AF) is the most common sustained arrhythmia encountered in clinical practice, with the highest prevalence observed among elderly people. Atrial fibrillation is responsible for markedly increased cardiovascular morbidity, and mortality and has been associated with various cardiovascular disorders, predominantly with hypertension, coronary artery disease, heart failure and valvular heart disease [1]. Various factors, including atrial remodeling and inflammation, have been implicated in the pathogenesis and perpetuation of AF; nevertheless the exact mechanism still remains uncertain [2-6]. Electrical remodeling is the possible substrate for persistence of AF after the initial event [7,8]. On the other hand, an underlying structural remodeling might occur before, during and after electrical remodeling, that is only in part reversible and can additionally contribute to AF maintenance [9].

Atrial structural remodeling is strongly connected with the fibrotic process and the subsequent disturbances in extracellular matrix (ECM) turnover. Matrix metalloproteinases (MMPs), a multi-gene family of structurally and functionally homogeneous proteolytic enzymes in balance with their tissue inhibitors (TIMPs), regulate ECM turnover and are proposed to have a determinant role in atrial structural remodeling involved in the development and perpetuation of AF [10-15]. Even though, levels of these markers have been shown to differ between AF and sinus rhythm (SR) individuals with impaired cardiac systolic function, there is limited knowledge regarding similar associations among patients with AF and patients with SR that have preserved left ventricular (LV) systolic function and a common cardiovascular disease substrate, such as essential hypertension.

In the present study we sought to investigate whether serum levels of MMP-2, MMP-3 and MMP-9 and their tissue inhibitor TIMP-1 differ in hypertensive patients with normal LV systolic function and different types of AF compared to their SR counterparts; we also evaluated associations of these markers with AF incidence and atrial structural remodeling. The latter was interpreted by measuring the left atrial volume (LAV) and LAV to body surface area (BSA) index ratio (LAV/BSA).

## Methods

### Study population

Before the initiation of any study procedures, a written informed consent was obtained from each study participant. The ethics committee of our institution approved the study, which was performed according to the principles outlined in the Declaration of Helsinki. The study was designed to be a nested case-control study within a prospective cohort of 175 consecutive patients with atrial fibrillation. Of those, 59 patients with established arterial hypertension and no other precipitated cardiovascular disorder or structural heart disease were included in the case-control analysis as cases. All patients were under anti-hypertensive treatment with angiotensin converting enzyme inhibitors (ACEIs) or angiotensin receptor blockers (ARBs) for at least a year from the moment of arterial hypertension diagnosis and none had diabetes, hyperlipidemia and a previous or current treatment with aldosterone receptor antagonists at the time of the study recruitment. Patients with conditions associated with elevated serum concentrations of myocardial or tissue fibrosis markers such as liver disease, renal impairment, pulmonary fibrosis and chronic obstructive pulmonary disease, extensive wounds, metabolic bone disease, malignancy, connective tissue disorders, chronic or acute inflammatory disease, or recent surgery were excluded from the study. Furthermore, patients over 80 years old and patients with a pacemaker/implantable cardioverter-defribrillator were also excluded.

In order to rule out conditions potentially associated with AF (such as coronary artery disease, heart failure, left ventricular hypertrophy, valvular heart disease, pericarditis, myocarditis and various cardiomyopathies), a standardized protocol including a detailed history, transthoracic echocardiography, exercise test and any other indicated test, was performed. The transthoracic echocardiogram was carried out using Hewlett Packard Sonos 2000 echocardiographic equipment.

Left ventricular dimensions [interventricular septal thickness (IVSDT), posterior wall thickness (PWDT), and LV internal diameter (LVIDD)] were measured at the end of diastole with M-mode by using the leading-edge to leading-edge convention. Left ventricular mass was determined by using the ASE formula according to the adjusted equation by Devereux et al [16]: left ventricular mass (g) = 0.8[1.04(LVIDD + IVSTD + PWTD)^3 ^- LVIDD^3^] + 0.6. Left ventricular mass was divided with the BSA area to obtain the LV mass index (LVMI). All patients with LVMI **≥ **150 g/m^2 ^were considered as having LVH, according to data from the Framingham Heart Study [17], and subsequently were excluded from our study.

Estimation of maximal LAV was performed as follows: from the apical four- and two-chambers view, left atrial borders were manually traced at end-systole. The biplane and single plane modified Simpson's formula was applied for maximal LAV measurement [18,19]. Images were stored in VHS magnetic tape and two independent observers performed the final estimation of LAV and LVMI off-line. Additionally, the LAV/BSA was used as a complementary way of assessing left atrial size, given that this method has been described as superior compared to the left atrial diameter or LAV alone in determining the existence of cardiovascular diseases [20].

### Laboratory methods and blood sampling

All patients of the AF group were in AF at the time of blood sampling. Blood samples, for the measurement of ECM turnover indices, were obtained by a peripheral vein draw and within an hour after collection they were centrifuged at 3,200 × g for 10 minutes at a temperature of about 4°C. The serum was separated into aliquots and was stored in -80°C until personnel blinded to the patients' clinical information performed the assay analysis. Serum levels of MMP-2, MMP-3, MMP-9 (pro- and active forms) and TIMP-1 were determined by commercial standardized in vitro enzyme-linked immunosorbent assay (ELISA) methods according to the manufacturer instructions (RayBiotech, Inc. USA). These assays detected only human MMPs and TIMPs with a sensitivity of < 140 pg/mL for MMP-2, < 0.3 ng/mL for MMP-3, < 10 pg/mL for MMP-9 and < 70 pg/mL for TIMP-1. The intra- and inter-assay coefficients of variation for all the assays were < 10% and < 12%, respectively.

### Statistical analysis

For the purposes of the analysis, the patients with AF were sub-divided into two groups. The first group consisted of subjects with symptomatic, paroxysmal AF of recent onset defined as spontaneously or pharmacologically terminated AF within a maximal period of less than 72 hours from arrhythmia onset. The latter was estimated based on patient recall of the onset of symptoms suggestive of AF, such as palpitations, shortness of breath and/or chest discomfort. The second group consisted of patients with permanent AF defined as sustained AF of more than six months in duration, without any documented intervening periods of SR that resisted to all attempts of SR restoration including pharmaceutical or direct current cardioversion. Several electrocardiograms that each patient underwent during their regular basis follow up confirmed the permanent AF. Electrocardiographic diagnosis of AF was made according to Bellet's definition [21]. In all AF subjects, rate control was achieved through utilization of beta-blockers or non-dihydropyridine calcium channel blockers and anti-coagulation therapy was administered as indicated. Finally, a third group of hypertensive patients with no history of AF or any other type of atrial arrhythmias and with the same cardiovascular profile as AF patients was used as a control group. Control patients and AF individuals were matched for LVMI and gender.

All quantitative variables were tested for normal distribution according to the Kolmogorov - Smirnov test. Normally distributed variables are expressed as mean and standard deviation values, whilst non - normally distributed data are presented as median and inter-quartile range values (IQR; 25th-75th percentile). Categorical variables were compared among the paroxysmal, permanent AF and control groups using the chi-square test for normally distributed variables and Fisher's exact test for non - normally distributed variables. One-way analysis of variance was used to test whether these three groups differed with respect to various continuous parameters of interest. A post-hoc analysis with Tukey tests was performed to identify individual levels of significance. If the homogeneity of variance assumption was violated, the non-parametric Kruskal -Wallis test and post - hoc analyses with Mann - Whitney U test were used instead, in order to spot differences in between the groups and individual levels of significance, respectively. Correlations were estimated through Spearman's rank correlation method. Univariate logistic regression analysis was performed to define factors significantly associated with AF incidence. Because the distribution of MMP-2, MMP-9 and TIMP-1 was skewed, logarithmic transformation of these variables was used in the analysis. Further examination of independent predictors for AF incidence was performed, by constructing a multivariate logistic regression model. In the latter, all variables that had a statistical significance of p < 0.25 during univariate logistic regression analysis were included. Values of p < 0.05 were considered statistically significant and all tests were two tailed.

Since the nature of our research protocol was exploratory, formal sample size calculations were performed with pilot data from this study. It was estimated that for a moderate delta effect of 0.85 in the mean levels of the measured markers within the three studied groups, a power of 80%, and a two-tailed alpha of 0.05, a minimum of 25 patients would be required for each arm of the study. Retrospective power calculations within the three groups revealed a high delta effect of 1.27, 1.42, 1.6 and 2.63 for MMP-2, MMP-3, MMP-9 and TIMP-9, respectively. These effects, for a two-tailed alpha of 0.05, corresponded to a power of more than 88.5% if more than 16 patients would be enrolled in each arm of the study.

## Results

### Study Cohort

The baseline clinical and demographic data of the study participants are presented in Table 1. Thirty-three patients had paroxysmal AF of recent onset, while 26 patients were in permanent AF. There were no significant differences between the AF groups regarding age, gender, systolic blood pressure (SBP), diastolic blood pressure (DBP), LVMI, ejection fraction and body mass index (BMI) (p = 0.848, p = 0.683, p = 0.717, p = 0.993, p = 0.343, p = 0.863 p = 0.639, respectively). However, patients with permanent AF had significantly higher LAV and LAV/BSA than those with paroxysmal AF (p < 0.001 for both comparisons). Control subjects were younger than patients with paroxysmal and permanent AF (p = 0.007 and p = 0.047, respectively), but they were comparable with regards to gender, BMI, LVMI, SBP and DBP (p = 0.262 and p = 0.501, p = 0.210 and p = 0.737, p = 0.859 and p = 0.988, p = 0.870 and p = 0.961, p = 0.826 and p = 0.783, respectively). In addition, patients with permanent AF had markedly increased LAV and LAV/BSA compared to controls (p < 0.001 for both comparisons); On the other hand, subjects with paroxysmal AF, had significantly higher LAV/BSA and a tendency of higher LAV compared to controls (p = 0.036, p = 0.077, respectively).

**Table 1:** Baseline characteristics and serum levels of MMP-2, MMP-3, MMP-9 and TIMP-1 in hypertensive patients with sinus rhythm, paroxysmal and permanent AF

*Variables*	*Sinus Rhythm (n = 27)*	*Paroxysmal AF (n = 33)*	*Permanent AF (n = 26)*	*P Value*
**Age ± SD (years)**	62.74 ± 7.20	69.51 ± 9.39	68.31 ± 8.25	***0.007***
**Gender (Males/Females)**	17/10	16/17	14/12	0.53
**BMI ± SD (kg/m^2^)**	29.78 ± 4.15	27.92 ± 4.05	28.92 ± 4.50	0.24
**LVMI ± SD (g/m^2^)**	96.10 ± 17.48	92.87 ± 19.77	95.13 ± 10.79	0.86
**LA volume ± SD (mm**^3^)	52.10 ± 13.50	63.27 ± 18.70	94.73 ± 25.14	***< 0.001***
**LA volume/BSA ± SD (mm^3^/m^2^)**	26.68 ± 7.32	33.81 ± 10.15	50.15 ± 14.43	***< 0.001***
**LVEF (%), median (IQR)**	65.0 (60.0-65.0)	62.0 (60.0-65.0)	61.0 (55.0-65.0)	***0.045***
**SBP (mmHg), median (IQR)**	132.67 ± 11.44	131.12 ± 13.446	133.54 ± 9.95	0.73
**DBP (mmHg), median (IQR)**	77.11 ± 5.76	78.05 ± 6.40	78.23 ± 6.07	0.77
**MMP-2 (pg/ml), median (IQR)**	37.00 (32.00-43.90)	206.20 (111.80-371.35)	36.23 (21.40-60.38)	***< 0.001***
**MMP-3 (ng/ml), median (IQR)**	10.61 ± 4.93	18.91 ± 4.77	17.52 ± 7.01	***< 0.001***
**MMP-9 (pg/ml), median (IQR)**	86.00 (34.00-232.50)	114.25 (55.25-187.53)	368.75 (275.63-575.00)	***< 0.001***
**TIMP-1 (ng/ml), median (IQR)**	3.06 (1.23-3.87)	0.58 (0.39-0.81)	0.42 (0.27-0.51)	***< 0.001***

BMI, body mass index; LVMI, left ventricular mass index; LA, left atrium; LVEF, left ventricular ejection fraction; SBP, systolic blood pressure; DBP, diastolic blood pressure; BSA, body surface area; SD, standard deviation; IQR, inter-quartile range

### Matrix metalloproteinases and their tissue inhibitors serum levels

**Figure 1:**
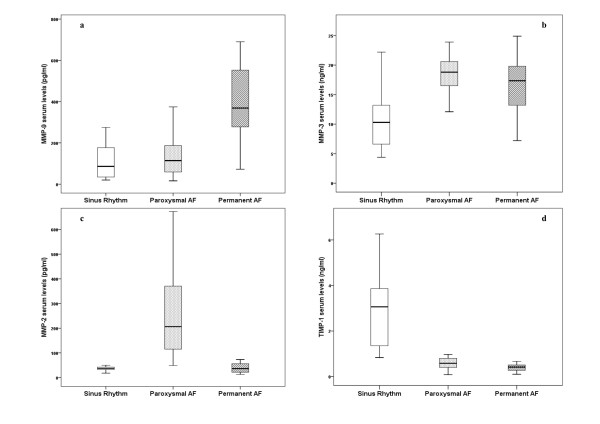
**Serum levels of MMP-9, MMP-3, MMP-2 and TIMP-1 in hypertensive patients with Sinus Rhythm (SR), paroxysmal and permanent AF**.

Patients with permanent AF had significantly higher serum levels of MMP-9 compared to both patients with paroxysmal AF and controls (p < 0.001 for both comparisons), whereas there was no significant difference between subjects with paroxysmal AF and those with SR (p = 0.6) (Figure 1a). Regarding MMP-3 serum levels, both patients with permanent and paroxysmal AF had markedly increased MMP-3 compared to controls (p < 0.001 for both comparisons), whilst there was no difference between AF groups (p = 0.6) (Figure 1b). Patients with paroxysmal AF had higher levels of MMP-2 when compared to patients with permanent AF or controls (p < 0.001 for both comparisons). On the other hand, MMP-2 values were comparable between subjects with permanent AF and controls (p = 0.625) (Figure 1c). Patients with permanent AF had lower levels of TIMP-1 than those with paroxysmal AF (p = 0.001) and the latter had lower levels of TIMP-1 than controls (p < 0.001) (Figure 1d).

**Figure 2:**
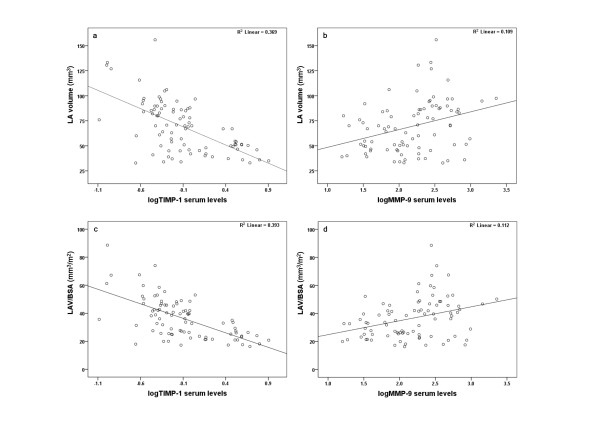
**Scatter dot diagrams, of all AF patients taken together, demonstrating direct linear correlations between: a**. left atrial volume and logMMP-9; **b**. left atrial volume and logTIMP-1; **c**. left atrial volume to BSA ratio and logMMP-9; **d**. left atrial volume to BSA ratio and logTIMP-1.

When all AF subjects were compared to those in SR, we observed that the latter had significantly lower serum levels of MMP-2, MMP- 3 and MMP-9 and higher TIMP-1 (Table 2). In addition, AF patients had significantly lower EF; they were older and had greater LAV and LAV/BSA than patients with SR (Table 2). Moreover, a strong inverse correlation between LAV and TIMP-1 levels (r = -0.396, p = 0.002) was found for AF patients (Figure 2a), whereas a positive correlation was noticed between LAV and MMP-9 levels (r = 0.451, p < 0.001) (Figure 2b). Likewise, LAV/BSA was inversely correlated with TIMP-1 (r = -0.414, p = 0.001) (Figure 2c) and positively correlated with MMP-9 (r = 0.443, p < 0.001) (Figure 2d). There were no significant correlations between the measured ECM turnover markers and ejection fraction, LVMI, age, systolic and diastolic blood pressure.

**Table 2:** Baseline characteristics and serum levels of MMP-2, MMP-3, MMP-9 and TIMP-1 in the total cohort of AF patients and individuals with sinus rhythm

*Variables*	*Sinus Rhythm (n = 27)*	*AF (n = 59)*	*P Value*
**Age ± SD (years)**	62.74 ± 7.20	68.98 ± 8.85	***0.002***
**Gender (Males/Females)**	17/10	30/29	0.295
**BMI ± SD (kg/m^2^)**	29.78 ± 4.15	28.36 ± 4.24	0.15
**LVMI ± SD (g/m2)**	96.10 ± 17.48	93.89 ± 16.14	0.65
**LA volume ± SD (mm**^3^)	52.10 ± 13.50	77.13 ± 26.71	***< 0.001***
**LA volume/BSA ± SD (mm^3^/m^2^)**	26.68 ± 7.32	41.01 ± 14.62	***< 0.001***
**LVEF (%), median (IQR)**	65.0 (60.0-65.0)	62.0 (60.0-65)	***0.02***
**SBP (mmHg), median (IQR)**	132.67 ± 11.44	132.19 ± 11.99	0.47
**DBP (mmHg), median (IQR)**	77.11 ± 5.76	78.13 ± 6.20	0.86
**MMP-2 (pg/ml), median (IQR)**	37.00 (32.00-43.90)	114.80 (37.20-338.35)	***0.001***
**MMP-3 (ng/ml), median (IQR)**	10.61 ± 4.93	18.30 ± 5.85	***< 0.001***
**MMP-9 (pg/ml), median (IQR)**	86.00 (34.00-232.50)	189.40 (95.90-361.90)	***0.011***
**TIMP-1 (ng/ml), median (IQR)**	3.06 (1.23-3.87)	0.47 (0.36-0.73)	***< 0.001***

BMI, body mass index; LVMI, left ventricular mass index; LA, left atrium; LVEF, left ventricular ejection fraction; SBP, systolic blood pressure; DBP, diastolic blood pressure; BSA, body surface area; SD, standard deviation; IQR, inter-quartile range

**Figure 3:**
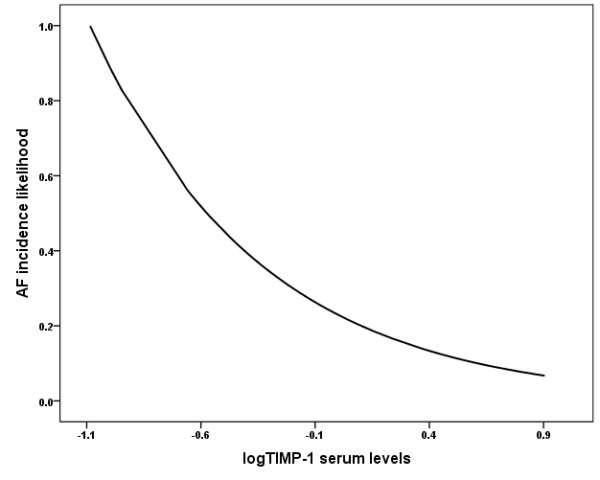
**The likelihood of AF incidence according to logTIMP-1 serum levels in patients with essential hypertension**.

Univariate analysis showed that lower logTIMP-1 was the strongest factor associated with AF incidence (OR: 0.257, 95%CI: 0.134-0.493, p < 0.001). Additional factors that were significantly associated with AF were MMP-3 levels (OR: 1.050, 95%CI: 1.015-1.086, p = 0.005); LAV (OR: 1.011, 95%CI: 1.002-1.020, p = 0.02); LAV/BSA (OR: 1.020, 95%CI: 1.004-1.036, p = 0.016). Further multivariate analysis adjusted for age, MMP-3, logMMP2, logMMP-9, ejection fraction, LAV and LAV/BSA showed that lower serum logTIMP-1 was the only independent factor associated with AF incidence (OR: 0.259, 95%CI: 0.104-0.645, p = 0.004), (Figure 3).

## Discussion

The transition of paroxysmal to persistent and eventually to permanent AF is a multi-factorial process. As implicated by the concept of "AF begets AF", AF per se forms the conditions for its relapse, persistence and perpetuation [7]. When arrhythmia becomes persistent, it is then associated with atrial enlargement and microscopic structural changes, both consisting of the main features of atrial structural remodeling [22,23]. Both the irregular ECM turnover and the fibrotic process are suggested to be important components of the latter [9,24-29].

The composition of ECM, which predominantly consists of collagen types I and III, fibril, fibronectin and laminin, is under the strict control of MMPs that are responsible for the degradation of all the matrix components between cells [30]. In addition, the TIMPs also play a significant role in this pathophysiological pathway by inhibiting the proteolytic activity of MMPs and both of them appear to be in a delicate balance [30]. Recently, it has been demonstrated that qualitative and quantitative changes in relevant ECM proteins and their regulatory molecules, might comprise a potent mechanism of AF. In particular, a study by Mukherjee et al. showed chamber specific differences of collagen content, MMPs and TIMPs levels in patients with heart failure and AF versus those in SR, suggesting a potential role for these molecules in the control of ECM remodeling in heart failure subjects with AF [12]. In their study, levels of MMP-1 in the right atrium, MMP-9 in the left atrium and TIMP-3 in the right ventricle, left atrium and left ventricle were greater with AF, whereas TIMP-1 levels were higher in the right ventricle of AF subjects. Furthermore, Xu et al showed that in the atria of patients with AF and end-stage heart failure there was increased deposition of collagen type I that was significantly associated with down-regulation of TIMP-2 and up-regulation of the activity and expression of MMP-2 [14]. These structural disturbances were subsequently associated with increased atrial dimensions and higher odds of AF recurrence [14]. Accordingly, patients with AF and relatively preserved LV systolic function had higher (as higher as the duration of AF was) activity of MMP-2 and MMP-9 and lower expression of TIMP-1. This deregulation was related to a considerably increased atrial fibrosis [15].

The results presented herein strengthen the aforementioned findings. In particular, we clearly demonstrated that lower serum TIMP-1 is the most powerful independent factor associated with higher AF incidence in patients with essential hypertension and normal left ventricular systolic function. Additionally, lower levels of TIMP-1 were strongly related to AF maintenance, whilst even though higher levels of MMPs - especially MMP-9 and MMP-3- were not an independent predictor of AF incidence, they were significantly increased in subjects with permanent AF. It appears that increased levels of MMPs with concomitant reduction of TIMP-1 may endorse atrial dilatation and AF persistence through the augmented breakdown of ECM [10]. On the other hand, this imbalance in favor of MMPs expression may be a part of a quite complex equation that might be related to collagen degradation products that are in a position to stimulate the development of a defectively structured fibrotic tissue [31]. Accordingly, our data demonstrated that increased MMP-9 and lower TIMP-1, were strappingly associated with increased left atrial remodeling as this was interpreted by LAV and LAV/BSA assessment. The exact mechanism behind the increase of MMP-9 in patients with fibrillating atria is unclear. Previous studies have shown that an ACE-dependent increase in the amount of activated extracellular signal-regulated kinases Erk1/Erk2 in atrial interstitial cells might stimulate the fibrotic process in atria with AF [32]. Angiotensin II has been implicated in the up-regulation of the MMP-9 and it has been showed that the former is associated with the cardiac hypertrophy process and cardiac remodeling [33]. It was also reported that angiotensin II is involved in the mechanism of the atrial electrical remodeling, and its blockade may lead to a better therapeutic management of AF [34]. Consequently, alterations in the renin-angiotensin system in AF subjects could substantiate a subsequent deregulation of MMPs secretion. In our study, however, all the participants were in anti-hypertensive treatment with ACE inhibitors or ARBs, suggesting that other additional mechanisms could trigger MMP-9 expression. Indeed, it has been shown that factors strongly associated with AF persistence involving inflammatory cytokines, increased transmural stretch and transforming growth factor beta-1 are potent inducers of MMPs expression including MMP-9 [35,36]

In contrast to the aforementioned results, others showed that in patients with AF, increased collagen deposition was associated with increased TIMPs to MMPs ratio [11]. This paradox was also the case in experimental studies with rapid atrial pacing induced AF, wherein increased MMP-9 expression was accompanied by an incremental tendency of TIMP-1 and -3 as well [37]. In addition, a recent study involving patients with lone AF revealed that individuals with persistent AF had higher serum levels of C-terminal pro-peptide of collagen type-I and TIMP-1 and lower levels of MMP-1 when compared to normal individuals [38]. These inconsistent results are quite intriguing and can be explained in part by the significant differences regarding the methodology of each study. In the first one, a model of rapid atrial pacing in pigs was used, demonstrating similar electrophysiological properties with human AF such as multiple re-entrant wavelets [39]. However, such experimental models do not relate closely to the most frequent clinical conditions associated with AF such as the cardiovascular disease substrate and consequential implications involving atrial dilatation, fibrosis and conduction slowing [40,41]. In the second study, a completely different cohort of patients with paroxysmal and persistent "lone" AF was used. Compared to our study population, these patients had notably higher levels of TIMP-1, were younger and had lower ejection fraction. The aging, the cardiovascular disease substrate and the functional capacity of the heart are factors that could be associated with ECM turnover and could affect the levels of proteins that regulate the latter. In our study, essential hypertension, properly treated with ACEIs or ARBs, was the underlying disorder for AF. Arterial hypertension or other heart disorders could have significant effects on the proteins that regulate ECM turnover and consequently the latter may have a diverse profile, strongly related to the precipitating cardiac diseases.

Another interesting finding of our study was the remarkably increased serum MMP-2 of patients with paroxysmal AF of recent onset compared to both, individuals with SR and those with permanent AF. It becomes evident that MMP-2 emerges as an acute reactant in recent onset AF. In fact, recent data [42-44] demonstrated that except for the extracellular activity of MMP-2 that occurs mainly on a "days to week" time scale, there is an acute MMP-2 up-regulation ("minutes to hours" time-scale) mediating the initial cellular response to enhanced oxidative stress. The latter appears to be associated with non-ECM protein targets. In particular, contrary to previous thoughts, MMP-2 has the capacity of myocardial intracellular expression, which can be actively up-regulated in response to several factors that have been involved in paroxysmal AF pathophysiology, including hypoxia, angiotensin II, endothelin-1 or pro-inflammatory cytokines [42,45,46]. In support of this, others showed that MMP-2, which is localized to the thin cardiac myofilaments, is implicated in myocardial stunning and ensuing contractile dysfunction during early ischemia/reperfusion injury through degradation of troponin I (TnI) and myosin light chain-1 [43,44]. Moreover, it has been observed that MMP-2 inhibitors may reduce TnI degradation in the myocardium after ischemia reperfusion injury process [43]. It could be considered, therefore, that MMP-2 can emerge as a molecule that can act on a far quicker time-scale in response to subtle cellular changes like those during initial stages of AF and contribute to early atrial stunning and contractile remodeling.

### Study limitations

Our results should be ideally confirmed by examination of additional markers of fibrosis, ECM remodeling and oxidative stress in tissue samples. For example, the additional assessment of the oxidative stress by measuring the serum levels of a marker like malondialdehyde would also add supplementary information with respect to the higher MMP-2 levels in patients with paroxysmal AF. Additionally, the immunoassay approach utilized in the present study could not differentiate the pro-form and active form of the MMP subtypes. Nonetheless, our principal purpose was not to examine the effect of MMPs to the fibrillating atria but rather to reveal any potential associations of different types of AF with specific MMPs patterns. Indeed, our findings showed a strong relation of AF with MMPs and that different subtypes of the latter could be associated with the different types of AF. Moreover, supplementary measurement of ECM turnover markers in several time intervals after the AF initiation could add significant information regarding their tendency throughout the initial stages of AF. However, the enrollment of AF patients with: essential hypertension as the only cardiovascular disease substrate; no echocardiographic evidence of structural heart disease (left ventricular hypertrophy); similar profile regarding the treatment of hypertension and our intensive efforts to exclude other factors associated with ECM remodeling contributed significantly to the reliability of our findings.

Due to insufficient data we could not further adjust for time since the first diagnosis of hypertension and time on anti-hypertensives, factors, that could have enhance our insight in the role of hypertension and structural heart effects and the associations with some of the observed changes in MMPs and TIMP-1. Additionally, even though we rigorously tried to match our study groups (AF and controls) with respect to all the referred demographic data, AF subjects were older than controls. However, paroxysmal and permanent AF groups were comparable for age and differed significantly with regards to MMP-2, MMP-9 and TIMP-1 levels. Additionally, there were no associations between age and ECM turnover makers in AF individuals, suggesting that age did not affect the interpretation of our results.

## Conclusions

In conclusion, we present data on serum TIMP-1 and MMPs levels in specific subsets of hypertensive patients with AF and SR. Lower serum levels of TIMP-1 were associated with increased AF incidence, whereas higher serum levels of MMP-3, MMP-9 and lower levels of TIMP-1 were strongly associated with permanent AF. Additionally, increased MMP-9 and lower TIMP-1 in patients with permanent AF were associated with higher states of atrial remodeling as this was assessed by LAV and LAV/BSA ratio, suggesting that the imbalance of TIMP-1 and MMP-9 could intensify atrial remodeling and subsequently lead to maintenance of AF. Finally, patients with AF of recent onset depicted markedly increased serum MMP-2 compared to both, control subjects and patients with permanent AF signifying a new potential role of MMP-2 as an acute reactant that might be linked with atrial dysfunction during the initial stages of AF.

## Competing interests

The authors declare that they have no competing interests.

## Authors' contributions

ASK has made critical and substantial contributions to the design and conception of this study. He also performed the statistical analysis of the data and significantly contributed to its acquisition, and interpretation. Finally, he had the leading role in the writing of the manuscript. ST has made substantial contributions to the design and conception of this study and significantly contributed to the writing of the manuscript. AGR has contributed to the design and conception of the study and the interpretation and analysis of the data. EAS has contributed to the collection, analysis and interpretation of the data and the design of the study. AT has contributed to the collection, analysis and interpretation of the data and the design of the study. DTK participated in the design and coordination of the study. IR conceived of the study and was the leader in its coordination. He also helped to draft the manuscript. All authors have read and approved the final version of the submitted manuscript.

## Pre-publication history

The pre-publication history for this paper can be accessed here:

http://www.biomedcentral.com/1471-2261/11/77/prepub
